# Rosmarinic Acid Exhibits Antifungal and Antibiofilm Activities Against *Candida albicans*: Insights into Gene Expression and Morphological Changes

**DOI:** 10.3390/jof10110751

**Published:** 2024-10-30

**Authors:** Merve Aydin, Nurhan Unusan, Esra Sumlu, Emine Nedime Korucu

**Affiliations:** 1 Department of Medical Microbiology, Faculty of Medicine, KTO Karatay University, Konya 42020, Turkey; 2 Department of Nutrition and Dietetics, Faculty of Health Sciences, KTO Karatay University, Konya 42020, Turkey; nurhan.unusan@karatay.edu.tr; 3 Department of Medical Pharmacology, Faculty of Medicine, KTO Karatay University, Konya 42020, Turkey; esra.sumlu@karatay.edu.tr; 4 Department of Molecular Biology and Genetics, Faculty of Science, Necmettin Erbakan University, Konya 42090, Turkey; enkorucu@erbakan.edu.tr

**Keywords:** rosmarinic acid, *Candida albicans*, antifungal activity, biofilm-related genes, FESEM

## Abstract

*Candida* species, opportunistic pathogens that cause various infections, pose a significant threat due to their ability to form biofilms that resist antifungal treatments and immune responses. The increasing resistance of *Candida* spp. and the limited availability of effective treatments have prompted the research of natural compounds as alternative therapies. This study assessed the antifungal properties of RA against *Candida* species, focusing on its impact on *C. albican*s biofilms and the underlying mechanisms. The antifungal efficacy of RA was evaluated using the CLSI M27-A3 microdilution method on both fluconazole-susceptible and -resistant strains. Biofilm formation by *C. albicans* was assessed through a crystal violet assay, while its antibiofilm activity was analyzed using an MTT assay and field emission scanning electron microscopy (FESEM). Gene expression related to biofilm formation was studied using quantitative real-time PCR (qRT-PCR), and statistical analysis was performed with an ANOVA. Among the 28 *Candida* strains tested, RA exhibited minimum inhibitory concentration (MIC) values ranging from 160 to 1280 μg/mL. At a 640 μg/mL concentration, it significantly reduced the expression of genes associated with adhesion (*ALS3*, *HWP1*, and *ECE1*), hyphal development (*UME6* and *HGC1*), and hyphal cAMP-dependent protein kinase regulators (*CYR1*, *RAS1*, and *EFG1*) in RAS1-cAMP-EFG1 pathway (*p* < 0.05). FESEM analysis revealed a reduction in hyphal networks and disruptions on the cell surface. Our study is the first to demonstrate the effects of RA on *C. albicans* adhesion, hyphae development, and biofilm formation through gene expression analysis with findings supported by FESEM. This approach distinguishes our study from previous studies on the effect of RA on *Candida*. However, the high MIC values of RA limit its antifungal potential. Therefore, more extensive research using innovative methods is required to increase the antifungal effect of RA.

## 1. Introduction

*Candida* (*C*.) species (spp.), a component of the microbiota in healthy individuals, are found on the skin and mucosal surfaces such as the oral cavity, gastrointestinal tract and vagina [1]. However, they can cause life-threatening opportunistic infections when the host immune system and gut microbiota are disrupted [2].

Despite the emergence of new and highly infectious *Candida* species in recent years, *C. albicans* remains the predominant causative organism of candidiasis [2,3]. However, the increasing prevalence of non-albicans species, particularly *C. parapsilosis*, *C. glabrata*, *C. tropicalis*, and *C. krusei*, presents significant challenges in diagnosis and treatment [3,4]. The rise of non-albicans *Candida* species is concerning due to their inherent resistance to certain antifungal agents. Some non-albicans species are naturally resistant to azoles and, to a lesser extent, echinocandins [5]. In addition, *C. glabrata* has a naturally lower susceptibility to azoles and can rapidly develop high resistance to azoles during treatment [6]. Furthermore, *C. tropicalis* and *C. parapsilosis* have been reported to be increasingly resistant to azoles. This evolving resistance pattern complicates the selection of effective antifungal treatments, as fluconazole resistance is particularly problematic in non-albicans *Candida* species [5,7,8]. The rapidly adaptive nature of *Candida* infections, coupled with marked regional differences in species distribution, emphasizes the need for novel targeted therapies to effectively manage the evolving epidemiology of *Candida* infections [9].

*Candida* spp., a polymorphic yeast, can exhibit various morphologies, including yeast, hyphae, and pseudohyphae forms, depending on environmental conditions [10]. The morphogenic plasticity (from yeast to hyphae), as well as the ability to form biofilms on abiotic surfaces, such as implanted medical devices, are important virulence factors that contribute to the pathogenesis of *Candida* spp. [11,12].

*Candida* species differ in their biofilm structure and characteristics. *Candida albicans* can produce denser biofilms than other species. It is considered to be the most important biofilm producer from a medical point of view [13]. *C. tropicalis* and *C. krusei* can produce true hyphae and pseudohyphae, whereas *C. parapsilosis* only produces pseudohyphae. In contrast, *C. glabrata* is not polymorphic and only forms biofilms as multi-layered structures or as clusters of yeast that form dense networks. These differences are important in understanding how *Candida* species contribute to clinical infections [12,13].

*C. albicans* biofilms are inherently resistant to most of the known antifungal drugs due to extracellular matrix production, upregulation of drug efflux pumps, and persistent cells, making the treatment of biofilm-associated infections a major challenge [14]. *C. albicans* biofilm infections can tolerate much higher concentrations of antifungal drugs than infections caused by planktonic cells [15]. *C. albicans* biofilms have been reported to exhibit up to 1000-fold higher resistance to fluconazole (FLC) and 10-fold higher resistance to amphotericin B compared to *C. albicans* planktonic cells [15,16].

The limited number of antifungal drugs currently available has many limitations, such as drug–drug interactions, poor pharmacokinetics, narrow therapeutic indices, and drug resistance as a result of their use [17]. Another concern is the rise in *Candida* strains resistant to nearly clinically available antifungals. Therefore, there is a need to develop novel antifungal therapies with novel modes of action [8,18].

In recent years, owing to the costly and time-consuming drug discovery process, there has been growing interest in natural pharmacological compounds as potential drug candidates. Natural products and derived compounds are important in drug discovery [19].

Polyphenolic acids are natural bioactive compounds that are found in almost all plants. Rosmarinic acid (RA, C18H16O8) is a well-known representative of this group, found in more than 160 species belonging to several families, including Lamiaceae, Boraginaceae, and *Apiaceae* [20]. Multiple phenolic hydroxyl groups are known to provide anti-oxidant activity to polyphenols. Rosmarinic acid has four phenolic hydroxyl groups, contributing to its potential anti-oxidant and antibacterial properties [20,21]. This characteristic has led to applications ranging from food preservation to cosmetics [22]. Owing to its ability to inhibit lipid peroxidation and bacterial growth, slow decay, and extend shelf life, RA has been approved for use in the food industry as a natural anti-oxidant and/or preservative [20,22].

Moreover, RA has demonstrated a broad spectrum of pharmacological activities, including anti-inflammatory, anti-oxidant, antibacterial, antiviral, antitumor, antidiabetic, neuroprotective, and hepatoprotective effects, as evidenced by numerous in vitro and in vivo studies [23].

Several studies have indicated that RA exhibits broad-spectrum antimicrobial activities, inhibiting both gram-positive and gram-negative bacteria, such as *S. aureus, L. monocytogenes*, *E. coli*, and *Klebsiella* spp. [24,25]. However, only a few studies have evaluated RA’s antifungal and antibiofilm efficacy [26,27,28].

We hypothesize that RA may affect virulence-related processes in *C. albicans* through multiple mechanisms. These mechanisms likely include the inhibition of adhesin proteins involved in host cell attachment and the disruption of hyphal formation pathways.

To the best of our knowledge, no studies to date have examined adhesion, hyphal, and biofilm-related genes to elucidate the antibiofilm effect of RA on *C. albicans*. In this regard, we aimed to investigate the antifungal and antibiofilm activities of RA against clinically isolated FLC-resistant and FLC-susceptible *Candida* species. Furthermore, we evaluated the effect of RA on biofilm formation, cell morphology and adhesion, yeast-hyphal transition, and expression levels of specific biofilm-associated genes involved in biofilm formation, offering insights into its antibiofilm capabilities.

## 2. Materials and Methods

### 2.1. Candida Strains

Our study included twenty-four clinical *Candida* strains from the Medical Mycology Laboratory Culture Collection of Gazi University Faculty of Medicine and four reference strains (*C. albicans* ATCC 10231, *C. glabrata* ATCC 90030, *C. krusei* ATCC 6258 and *C. parapsilosis* ATCC 22019). These strains were identified both phenotypically and genotypically. All strains were in glycerol storage at −80 °C and were subcultured on Sabouraud dextrose agar (Neogen Corporation, Lansing, MI, USA) and incubated overnight at 37 °C prior to testing.

### 2.2. Antifungal Susceptibility Testing

The antifungal activity of RA (AmBeed, Arlington, IL, USA) and FLC (ChemCruz, Dallas, TX, USA) against all strains of *Candida* was determined by a broth microdilution method according to the CLSI-M27-A3 method, as previously reported [29]. In brief, RA and FLC were tested at 5 to 2560 μg/mL and 0.5 to 256 μg/mL, respectively. The *Candida* cell suspensions were adjusted to an inoculum of 10^6^ cells/mL in an RPMI 1640 medium (Gibco, Billings, MT, USA) buffered with MOPS (AppliChem, Darmstadt, Germany), added to the 96-well microplate, and incubated at 37 °C for 24 h. The minimum inhibitory concentration (MIC) values were considered the lowest concentration of visual readings of fungal growth.

### 2.3. Biofilm Formation and Inhibition

To evaluate the biofilm ability of *C. albicans*, biofilm mass was determined using the Crystal Violet (CV) staining method as previously described [30]. For this purpose, cell suspensions (10^6^ cells/mL) in an RPMI 1640-MOPS medium were prepared from *C. albicans* overnight cultures, transferred to a 96-well microplate, and incubated at 37 °C for 24 h. Then, the supernatant was discarded, and the biofilm was rinsed with 1 × PBS (AppliChem, Germany) to remove non-adherent cells. After drying the microplate at 60 °C for 30 min, the biofilm was stained with 50 μL of a 1% *w/v* CV solution (Carlo Erba Reagents, Milan, Italy) and incubated for 15 min. The plates were washed twice with 1 × PBS buffer, and 150 μL of absolute ethanol was added to dissolve the biofilm-bound CV solution. The absorbance of the microplates was measured at 590 nm using a microplate reader (Multiskan Sky, Thermo Fisher Scientific, Waltham, MA, USA). All samples were performed in three replicates. According to the classification of Stepanovic et al., the isolates were divided into four levels of adhesion: non-adherent, weakly adherent, moderately adherent, and strongly adherent [31].

The antibiofilm activity of RA was evaluated using an MTT reduction assay at the biofilm formation stage, as previously described by our group [30]. The formation of biofilms was conducted using the CV staining method above. Briefly, *Candida* cell suspension (200 μL, 10^6^ cells/mL) was transferred into 96-well microplates and incubated at 37 °C for 90 min to allow adhesion formation. Following the adhesion phase, 200 μL of RA and FLC in a two-fold dilution series (prepared in RPMI 1640-MOPS medium for a final concentration range of 5–2560 μg/mL and 0.5–256 μg/mL, respectively) were transferred to the wells of the microtiter plates and incubated at 37 °C for a further 24 h. At the end of the process, the supernatant was removed, the biofilm was rinsed twice with 1 × PBS, and 20 μL of an MTT solution (5 mg/mL; Bio Basic, Toronto, ON, Canada) was added to each well containing treated and untreated biofilms and incubated for 4 h at 37 °C in darkness. Following removing the supernatant, 100 μL of DMSO (Thermo Fisher Scientific, Waltham, MA, USA) was added to each well and incubated for 10 min at 37 °C. Absorbance was measured at 570 nm using a MultiSkan Sky Microplate Reader. The inhibition percentages were calculated as % biofilm inhibition = Abs control − Abs sample/Abs control × 100. The experiments were carried out in triplicate.

### 2.4. The Effect of Rosmarinic Acid on Adhesion, Hyphae and Biofilm-Related Gene Expression

Quantitative gene expression analysis was performed to elucidate the antibiofilm mechanisms of RA on *C. albicans*, as previously described [30]. Briefly, *C. albicans* ATCC 10231 and two strongly adherent clinical strains (C1 and C7) were inoculated in RPMI-1640 medium in a 150 mm culture dish (SPL Life Sciences, Pocheon, Republic of Korea). After 90 min, to allow for cell adhesion, the wells were washed with PBS to remove non-adherent cells. Cells were incubated for 24 h at 35 °C with shaking at 75 rpm in the presence or absence of RA (640 µg/mL) and FLC (8 µg/mL). After that, biofilms were gently washed three times with 1 PBS to remove non-adherent cells and collected using a sterile cell scraper.

According to the manufacturer’s instructions, total RNA was extracted from the *C. albicans* biofilms using the YeaStar RNA Kit (Zymo Research, Orange, CA, USA). The concentration and purity of the extracted RNA were quantified using μDrop Plate Spectrophotometer (Thermo Fisher Scientific, USA). Then, one µg of total RNA was reverse transcribed into cDNA with the iScript cDNA synthesis kit (Bio-Rad, Hercules, CA, USA). Gene-specific primers were used to evaluate the expression levels of adhesion, hyphal, and biofilm-related genes (*ALS3*, *HWP1*, *ECE1*, *UME6*, *HGC1*, *RAS1*, *CYR1*, and *EFG1*) as listed in Table 1.

The experiment was carried out in a final volume of 20 µL containing 10 µL of 2× SYBR Green Master Mix (Roche, Basel, Switzerland), 1 µL of each primer at 10 pmol/µL, 5 µL of cDNA template, and 3 µL of nuclease-free water. Quantitative real-time PCR (qRT-PCR) was performed and analyzed using a QuantStudio™ 3 (Thermo Fisher Scientific, Waltham, MA, USA) with the following cycling conditions: 10 min at 95 °C, followed by 40 cycles of denaturation at 95 °C for 10 s and extension at 72 °C for 15 s. The experiments were performed in triplicates. The relative expression level of the target genes was normalized to 18S rRNA, and the fold change in gene expression was calculated using the 2^−∆∆^Ct method.

### 2.5. Field Emission Scanning Electron Microscopy (FESEM)

The effect of RA on *C. albicans* biofilm formation and cell morphological changes was analyzed using FESEM as described previously [30]. Accordingly, special plastic coverslips (SPL Life Sciences, Republic of Korea) were placed in 12-well cell culture plates, and a *C. albicans* ATCC 10231 cell suspension was added and incubated at 37 °C for 90 min. After adherence, the coverslips were incubated for 24 h in the presence or absence (untreated controls) of RA (640 µg/mL) and FLC (8 µg/mL). The slides were gently washed with 1 × PBS to remove loosely adherent cells and fixed with 2% (*v/v*) glutaraldehyde (Tekkim, Bursa, Turkey) for 1 h at 25 °C. Fixated cells were dehydrated using a graded ethanol series (10, 25, 50, 75, 90, and 100%) and then allowed to air-dry. The dried coverslips were mounted onto stubs and subsequently sputter-coated with iridium using a high-vacuum coating device (Leica EM ACE600, Wetzlar, Germany). Finally, the coated coverslips were examined under a field emission scanning electron microscope (ZeissGemini500, Oberkochen, Germany) at magnifications varying from 500× to 5000×.

### 2.6. Statistical Analysis

GraphPad Prism 9 (GraphPad Software, Inc., La Jolla, CA, USA) was used for statistical analyses. The differences between the groups were evaluated using an analysis of variance (ANOVA) and Bonferroni post hoc test. The data, presented as mean + standard error mean (SEM), revealed significant findings. *p* values less than 0.05 were defined as statistically significant.

## 3. Results

### 3.1. Antifungal Activity

The antifungal activity of rosmarinic acid was determined using minimum inhibitory concentration (MIC) in 4 standard strains and 28 clinical *Candida* isolates. The MIC value range is given in Table 2, and the MIC values of each strain are detailed in Supplementary Table S1.

The antifungal activity of RA in *C. albicans* 10231 and *C. parapsilosis* 22019 was similar to their clinical isolates with an MIC of 640 μg/mL. However, clinical isolates of both species were slightly more resistant to RA with MICs of 640–1280 μg/mL. The MIC values of *C. glabrata* 90030 and *C. krusei* 6258 for RA were 160 and 320 μg/mL, respectively. Interestingly, both species were more sensitive to RA than *C. albicans*, the most common strain. The MIC for clinical *C. glabrata* strains was 160–320 μg/mL, similar to the reference. However, the MIC value for clinical *C. krusei* isolates was 1280 μg/mL, considerably higher than the MIC value of the reference strain. The rosmarinic acid MIC values for *C. kefyr* isolates were 160–1280 μg/mL, while the MIC values for *C. lusitaniae* isolates were 640–1280 μg/mL. These results showed that RA can be broadly effective against *Candida* species in the range of 160–1280 μg/mL.

### 3.2. Biofilm-Forming Capacity and Anti-Biofilm Activity

**Figure 1:**
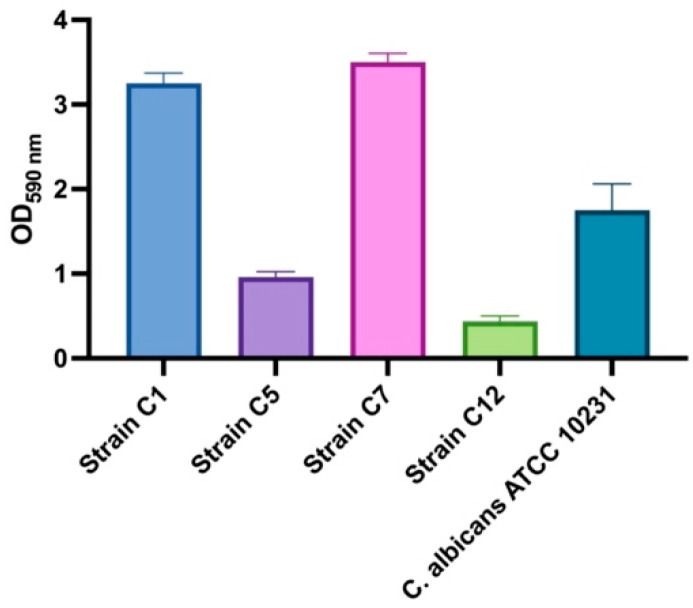
OD_590 nm_ values of *C. albicans* biofilms in CV biofilm assay after 24 h.

Biofilm formation capacity, the most important virulence factor of *C. albicans*, was determined using a CV biofilm assay in four clinical and one reference strain. According to the classification of Stepanovic et al. [31], the cut-off OD (ODc) value was determined by averaging the OD values of the negative control wells. An ODc < OD ≤ 2 × ODc was considered a weak biofilm, a 2 × ODc < OD ≤ 4 × ODc was considered a moderate biofilm, and a 4 × ODc < OD was considered a strong biofilm. In our experiment, the ODc value was calculated as 0.4256. All *C. albicans* strains used in the study were found to be capable of producing a biofilm. The OD values of the strains are shown in Figure 1. Of the four clinical strains, two formed a strong (Strains C1 and C7), one a medium (Strain C5), and one a weak (Strain C12) biofilm. Therefore, *C. albicans* ATCC 10231, strains C1 and C7, with strong biofilm ability, were selected to determine the antibiofilm activity of RA.

**Figure 2:**
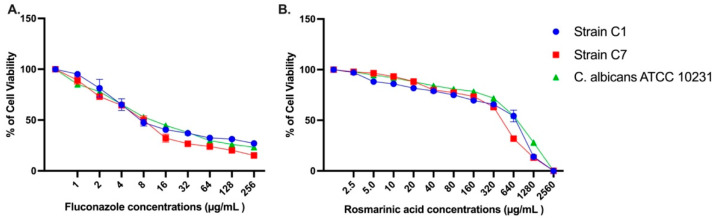
Percentage of cell viability of *C. albicans* biofilms treated with (**A**) Fluconazole, (**B**) Rosmarinic acid at 24 h by MTT test.

An MTT assay was performed to examine the metabolic activity of RA in biofilms. Three strains with strong biofilms (*C. albicans* ATCC 10231, Strains C1 and C7) were exposed to RA and FLC ranging from 2.5 to 2560 µg/mL and 1 to 256 µg/mL, respectively, for 24 h. In the MTT assay, RA at a 640 µg/mL concentration decreased the metabolic activity in *C. albicans* biofilms. Therefore, the minimum inhibitory concentration of RA was 640 µg/mL in further experiments. The experiments were repeated for FLC, and the MBIC value was found to be 8 µg/mL. Metabolic activity results of *C. albicans* ATCC 10231, C1, and C7 strains are presented in Figure 2.

### 3.3. Biofilm-Related Gene Expressions

**Figure 3:**
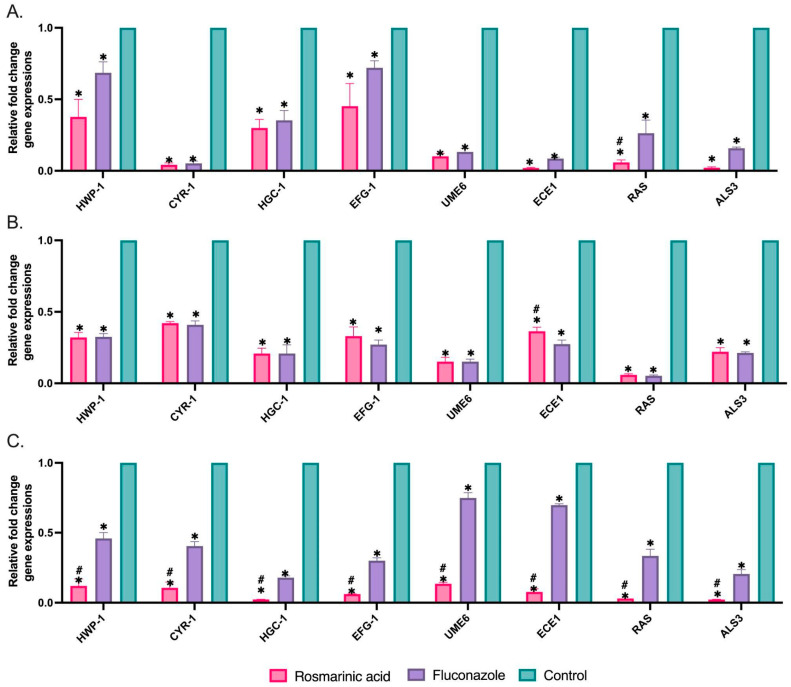
Effect of rosmarinic acid on the expression of biofilm-related genes in (**A**) strain C1, (**B**) strain C7, and (**C**) *C. albicans* ATCC 10231. *18S rRNA* used for normalization of gene expression levels. Values are expressed as mean ± SEM, * *p* < 0.05, significantly different from the control; # *p* < 0.05, significantly different from the fluconazole.

To elucidate the molecular mechanisms underlying biofilm formation and its key stages, such as adhesion and hyphal development, we evaluated transcriptional changes in eight specific genes (*ALS3*, *HWP1*, *ECE1*, *UME6*, *HGC1*, *RAS1*, *CYR1*, and *EFG1)* using qRT-PCR, as shown in Figure 3. This analysis was performed on strong biofilm-forming strains (*C. albicans* ATCC 10231, strain C1, and strain C7) exposed to MBIC50 concentrations of RA and FLC. Compared to the control group, the expression of the genes we examined was significantly altered in RA-treated biofilm cells. Moreover, the changes in gene expression induced by RA were similar to those in FLC-treated cells. Compared to FLC, RA significantly reduced *ECE1* in strain C7 and all genes examined in the reference strain. Considering these results, the antibiofilm effect of RA determined at 640 μg/mL may be related to altered biofilm-associated genes.

### 3.4. FESEM Analysis

**Figure 4:**
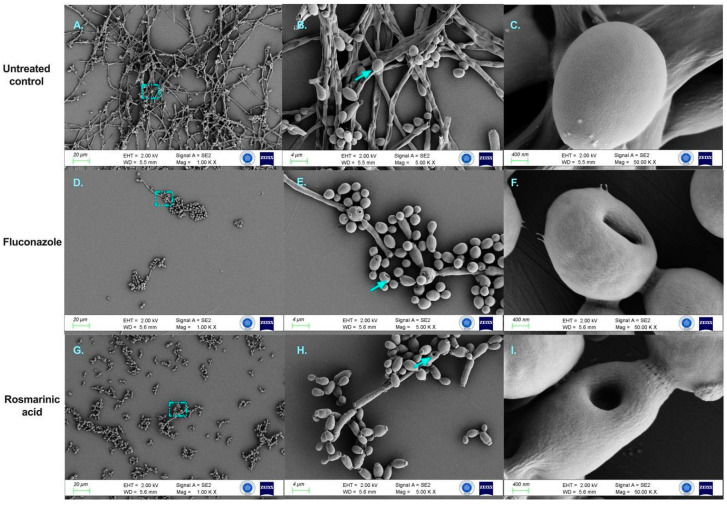
FESEM images of *C. albicans* ATCC 10231 biofilms after control and 24 h exposure to rosmarinic acid and fluconazole. (**A**–**C**). Untreated control biofilm culture. (**D**–**F**). Biofilm culture treated with 640 μg/mL rosmarinic acid. (**G**–**I**). Biofilm culture treated with 8 μg/mL fluconazole. Magnification scales of 1000×, 5000×, and 50,000× were used for imaging. Blue arrows indicate unusual surfaces and pore formation. The blue frame shows the selected region.

FESEM analysis observed the morphological changes of RA in *C. albicans* mature biofilms, as shown in Figure 4. Untreated cell surfaces were smooth and clustered with dense mycelial cross-links, with yeast and hyphae coexisting. Rosmarinic acid-treated cells showed relaxed mycelia and pores on the surface of yeast cells, similar to those treated with FLC. In addition, yeast-like cells were significantly increased in the RA group, similar to fluconazole. According to these results, it was determined that RA can significantly disrupt biofilm architecture.

## 4. Discussion

Evidence suggests that the antimicrobial properties of rosemary extracts are closely linked to its essential oils and phenolic compounds, such as RA. Phenolic compounds can exhibit antimicrobial effects through mechanisms including DNA damage, interference with transcription, inhibition of enzymatic activity, and regulation of gene expression related to virulence factors [32]. Moreover, polyphenols can act as pro-oxidants by generating hydrogen peroxide, contributing to their antimicrobial activity [33]. Furthermore, natural polyphenols have been indicated as promising molecules against *C. albicans* [34].

A limited number of studies have demonstrated the antifungal properties of RA on *Candida* spp. [26,27,28]. In a previous study, RA inhibited growth against *Candida albicans* ATCC 10231 at 1000 μg/mL concentration. This anticandidal activity of RA is attributed to its ability to inhibit isocitrate lyase, a key enzyme involved in the glyoxylate cycle of *C. albicans* [26]. Bittner Fialová et al. evaluated the antimicrobial activity of RA and its newly synthesized derivatives, demonstrating that RA had an MIC of 1.73 mM for *Candida albicans* CCM 9002. In the same study, RA-derivative phosphonium salts showed enhanced antifungal activity [27]. Another recent study reported that RA exhibited promising anticandidal activity (MIC 0.1–0.2 mg/mL) in reference to clinical *Candida* isolates obtained from tonsillar tissue and oral cavities of patients. However, *C. albicans* ATCC 10231, *C. krusei* H1/16, and *C. tropicalis* ATCC 750 were more resistant to RA (MIC 0.2 mg/mL) than clinical isolates [28].

In our study, RA was shown to be effective against clinical and reference strains of *Candida* species such as *C. albicans*, *C. glabrata*, *C. krusei*, *C. lusitaniae,* and *C. kefyr* in the range of 160–1280 μg/mL.

Conversely, some studies have reported that RA showed limited or no antimicrobial activity, but these findings were typically based on a narrow MIC range [35]. Our study utilized a wider MIC range of 5–2560 μg/mL and revealed the anti-candidal activity of RA at a concentration of 1280 μg/mL. This discrepancy in results may be attributed to the different MIC ranges used, which could lead to inconsistent findings regarding the activity of RA against *Candida* species.

Only a few studies have investigated RA adhesion and biofilm formation on various *Candida* species. Ivanov et al. found that RA effectively inhibited adhesion of *C. albicans* strain 475/15 and moderately affected *C. albicans* ATCC 10231. The adhesion of non-albicans *Candida* strains was reported to be more significantly affected by RA compared to *C. albicans*, except for *C. tropicalis.* In the same study, RA at 0.4 mg/mL inhibited biofilms formed by *C. albicans* 475/15 and *C. albicans* ATCC 10231. Biofilms of *C. krusei* H1/16 were reported to be the most resistant to RA at MBIC >1.6 mg/mL. Unlike cell adhesion, RA-affected biofilms formed by *C. albicans* strains more than nonalbicans *Candida*. Similar to this study [28], our study found the MBIC value of RA for *C. albicans* biofilms to be 640 μg/mL by an MTT assay.

To our knowledge, no comprehensive studies are elucidating the molecular mechanisms underlying the potential anti-biofilm activity of RA and its impact on the transcriptional changes of biofilm development. Rosmarinic acid exhibits potential anti-biofilm activity against *C. albicans* at a dose of 640 µg/mL through multiple mechanisms, as revealed by our study using qRT-PCR and FESEM studies. RA significantly reduced the expression levels of *RAS1*, *CYR1*, and *EFG1*, which are crucial components of the Ras1-cAMP-Efg1 pathway. The Ras1-cAMP-Efg1 pathway is the main signaling pathway that regulates adhesion, yeast-to-hyphae transition, and biofilm formation in *C. albicans* [36]. *RAS1* is a GTPase that induces adhesion and hyphae formation by activating *CYR1* adenylate cyclase. *CYR1* activates the key transcription factor *EFG1*, which regulates several genes important in filamentous growth and biofilm formation [36,37].

Furthermore, the expression of key transcription factors downstream of this pathway, *HGC1* and *UME6*, was markedly suppressed by RA treatment. Hyphal growth involves several transcriptional factors, with *UME6* and *HGC1* playing crucial roles in the early stages of filament formation [38]. *HGC1* has been shown to affect the activity of two other key transcriptional regulators of hyphal growth, *EFG1* and *UME6* [39]. Previous research indicates that *HGC1* is activated before *UME6*. However, *UME6* regulates the level and duration of *HGC1* expression, which is essential for elongation [39,40]. RA treatment also significantly downregulates genes encoding adhesins, such as *ALS3* and *HWP1*. These adhesins are critical for the initial attachment of *C. albicans* cells to surfaces and cell-cell interactions within the biofilm [41]. Moreover, the expression of *ECE1*, a gene linked to adhesion, biofilm formation, and the yeast-to-hypha transition [42], was also significantly suppressed by RA.

In addition, RA and fluconazole showed similar efficacy in clinical strains, whereas RA significantly suppressed gene expression in the reference strain compared to fluconazole.

The morphological and structural alterations induced by rosmarinic acid (RA) on *C. albicans* were examined using field emission scanning electron microscopy (FESEM). Untreated *C. albicans* cells exhibited a characteristic cylindrical morphology with smooth surface features and an intricate hyphal network interconnecting yeast and hyphal forms. In contrast, *C. albicans* cells exposed to RA (640 μg/mL) or fluconazole (FLC, 8 μg/mL) displayed significant morphological changes, including loss of surface smoothness and the formation of atypical pores. Furthermore, treatment with RA or FLC resulted in a marked reduction of the hyphal network, with a concomitant increase in yeast cell predominance. This shift in cellular morphology suggests a disruption of the balance between yeast and hyphal forms, which is crucial for biofilm formation and maintenance. The alterations in cell surface integrity and hyphal network formation indicate that RA deleterious affects *C. albicans* biofilm structure.

The effect of RA treatment on gene expression is confirmed by the reduction of biofilm formation in vitro observed with FESEM. Furthermore, our results suggest that the inhibition of hyphae/biofilm-related genes is probably mediated by Ras1-cAMP-Efg1. This finding further highlights the multi-faceted approach by which RA inhibits *C. albicans* biofilm formation.

Our study has several limitations. While we investigated the antifungal activity of RA in 28 strains, including *C. albicans*, *C. lusitaniae*, *C. glabrata*, *C. krusei*, *C. kefyr*, *C. parapsilosis,* and reference species, we could not specifically evaluate the antibiofilm activity of RA in non-albicans strains. The antibiofilm activity of rosmarinic acid may vary among different *Candida* species due to differences in biofilm structure. Future studies should investigate this aspect to understand better RA’s potential among various species and biofilm structures. Furthermore, obtaining an effective dose of RA, a secondary metabolite, under in vivo conditions may pose significant challenges due to its poor bioavailability. Given the limitations of our study, more extensive preclinical and clinical studies involving experimental animals are needed to demonstrate the antifungal and anti-biofilm effects of RA.

## 5. Conclusions

In conclusion, our study demonstrates that RA exerts its anti-biofilm effect against *C. albicans* by targeting multiple key pathways and genes involved in adhesion, hyphal growth, and biofilm development. The multiple phenolic hydroxyl groups present in RA’s structure may mediate these effects.

Despite the promising antibiofilm properties exhibited by rosmarinic acid (RA), its therapeutic potential as an antifungal is constrained by the relatively high MIC values observed in our study. Future research directions may include investigating synergistic combinations with antifungal agents, developing novel drug delivery systems, or investigating structural modifications to enhance its antifungal activity.

## Figures and Tables

**Table 1:** List of primers used in this study.

Genes	Primer	Sequence (5′→3′)
*HWP1*	Forward	GCTCCTGCTCCTGAAATGAC
	Reverse	CTGGAGCAATTGGTGAGGTT
*CYR1*	Forward	CCAACAAACGACCAAAAGGT
	Reverse	TCTTGAACTGCCAGACGATG
*HGC1*	Forward	GCTTCCTGCACCTCATCAAT
	Reverse	AGCACGAGAACCAGCGATAC
*EFG1*	Forward	GCCTCGAGCACTTCCACTGT
	Reverse	TTTTTTCATCTTCCCACATGGTAGT
*UME6*	Forward	ACCACCACTACCACCACCAC
	Reverse	TATCCCCATTTCCAAGTCCA
*ECE1*	Forward	TTGCTAATGCCGTCGTCAGA
	Reverse	GAACGACCATCTCTCTTGGCAT
*RAS1*	Forward	TGGATGTTGTGTTATTGTTTGAGC
	Reverse	GTCTTGAATTGTTCATCTTCTCCCA
*ALS3*	Forward	TCGTCCTCATTACACCAACCA
	Reverse	TGAAGTTGCAGATGGGGCTT
*18S rRNA*	Forward	AGAAACGGCTACCACATCCA
	Reverse	AGCCCAAGGTTCAACTACGA

**Table 2:** MIC range of fluconazole (FLC) and rosmarinic acid (RA) to *Candida* isolates.

Species (Number)	MIC_50_ Range of FLC (μg/mL)	MIC_50_ Range of RA (μg/mL)
*C. albicans* [4]	1–4	640–1280
*C. lusitaniae* [4]	2–64	640–1280
*C. glabrata* [4]	16–32	160–320
*C. krusei* [4]	32	1280
*C. kefyr* [4]	1–4	160–1280
*C. parapsilosis* [4]	2–64	640–1280
*C. albicans* ATCC 10231	1	640
*C. glabrata* ATCC 90030	16	160
*C. krusei* ATCC 6258	64	320
*C. parapsilosis* ATCC 22019	2	640

## Data Availability

The raw data supporting the conclusions of this article will be made available by the authors on request.

## Supplementary Materials

The following supporting information can be downloaded at https://www.mdpi.com/article/10.3390/jof10110751/s1, Table S1: MIC value for fluconazole (FLC) and rosmarinic acid (RA) against *Candida* species in detail. Figure S1: Percentage of cell viability of *C. albicans* biofilms through the MTT assay at 24 h in detail; (A,D) strain C1, (B,E) strain C7, and (C,F) *C. albicans* ATCC 10231.
