# Supplementary material for: Rosmarinic Acid Exhibits Antifungal and Antibiofilm Activities Against Candida albicans: Insights into Gene Expression and Morphological Changes
Source: J Fungi (Basel). 2024 Oct 30;10(11):751. doi: 10.3390/jof10110751 (PMC11595412; doi:10.3390/jof10110751)

Supplementary Table S1: MIC value for fluconazole (FLC) and rosmarinic acid (RA) against *Candida* species in detail.

No	Strain	Fluconazole (μg/ml)	Rosmarinic Acid (μg/ml)
C1	*C. albicans*	1	640
C5	*C. albicans*	4	640
C7	*C. albicans*	2	1280
C12	*C. albicans*	2	640
	C. albicans 10231	1	640
C10	*C. lusitaniae*	4	1280
C33	*C. lusitaniae*	2	1280
C37	*C. lusitaniae*	64	1280
C38	*C. lusitaniae*	32	640
C21	*C. glabrata*	16	160
C22	*C. glabrata*	32	320
C23	*C. glabrata*	32	320
C24	*C. glabrata*	32	320
	C. glabrata ATCC 90030	16	160
C15	*C. krusei*	32	1280
C16	*C. krusei*	32	1280
C17	*C. krusei*	32	1280
C19	*C. krusei*	32	1280
	C. krusei ATCC 6258	64	320
C11	*C. kefyr*	2	1280
C13	*C. kefyr*	2	1280
C14	*C. kefyr*	1	320
C32	*C. kefyr*	4	160
C34	*C. parapsilosis*	64	1280
C35	*C. parapsilosis*	8	1280
C41	*C. parapsilosis*	2	640
C42	*C. parapsilosis*	32	640
	C. parapsilosis ATCC 22019	2	640

According to CLSI, a strain is considered resistant when the fluconazole dose is ≥8 ug/ml for *C. albicans* and *C. parapsilosis* and ≥64 ug/ml for *C. glabrata*. A strain is also considered susceptible-dose dependent (SDD) when the dose is 4 ug/ml for *C. albicans* and *C. parapsilosis* and ≤32 ug/ml for *C. glabrata. C. krusei* is intrinsically resistant to fluconazole. EUCAST's non-species-associated breakpoint value for fluconazole was used. Accordingly, ≤2 was considered sensitive and >4 mg/L resistant. Based on this information, our collection has 6 SDD and 10 resistant strains. Of note, most of the clinical isolates were resistant to fluconazole.

Supplementary Figure S1. Percentage of cell viability of *C. albicans* biofilms through the MTT assay at 24 h in detail; A, D) strain C1, B, E) strain C7, and C, F) *C. albicans* ATCC 10231.

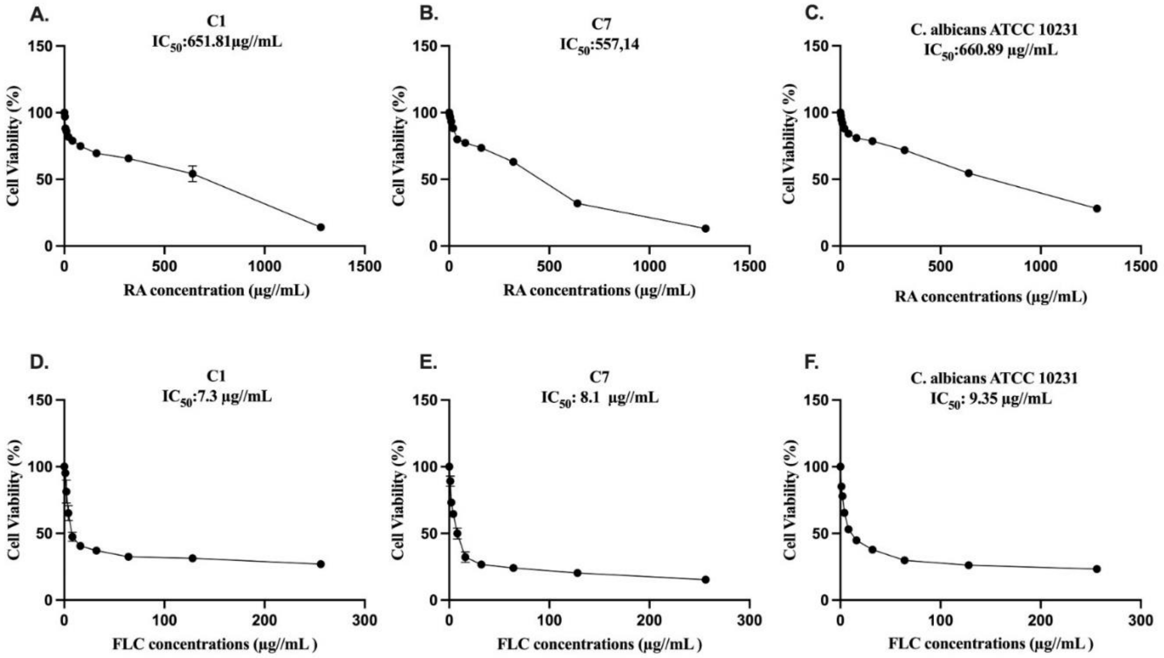

Supplement: Supplementary file 1 [file jof-10-00751-s001.zip › jof-3233039-supplementary.pdf]
